# Revisiting the hematological manifestations of vitamin B12 deficiency

**DOI:** 10.3389/fnut.2026.1898533

**Published:** 2026-08-12

**Authors:** Nupur Parakh, Pooja Dewan

**Affiliations:** 1 Department of Hematology, Bone Marrow Transplantation and Cellular Therapy, Vardhman Mahavir Medical College and Safdarjung Hospital, Guru Gobind Singh Indraprastha University, New Delhi, India; 2 Department of Pediatrics, University College of Medical Sciences, University of Delhi, New Delhi, India

**Keywords:** cobalamin, folate, megaloblast, methylcobalamin, pancytopenia

## Abstract

Vitamin B12 (cobalamin) deficiency remains a significant problem affecting both children and adults and is associated with a wide range of hematological manifestations that are largely preventable with timely diagnosis and treatment. Vitamin B12 deficiency can present with variable hematological, neuropsychiatric, and mucocutaneous changes. Hematological abnormalities are among the most common presentations. The classical peripheral blood smear shows macrocytosis with oval macrocytes, anisopoikilocytosis, and hypersegmented neutrophils. Anemia may be accompanied by leukopenia and thrombocytopenia due to ineffective hematopoiesis. Bone marrow examination typically reveals hypercellularity with erythroid hyperplasia and large megaloblasts exhibiting open chromatin and nuclear–cytoplasmic asynchrony as a result of defective DNA synthesis. Ineffective erythropoiesis leads to intramedullary destruction of hematopoietic precursors, resulting in elevated lactate dehydrogenase, indirect hyperbilirubinemia, and reduced haptoglobin, thereby mimicking hemolytic anemias. Abnormal red cells and megaloblasts that enter the circulation have a shortened lifespan, leading to megaloblastic anemia, with reticulocyte counts that are inappropriately low for the degree of anemia. In pediatric patients, manifestations may be severe, particularly in infants born to cobalamin-deficient mothers. Presentations include profound anemia, pancytopenia, failure to thrive, recurrent infections, and bleeding manifestations due to thrombocytopenia, often misdiagnosed as aplastic anemia or myelodysplasias. Pigmentary changes in the form of hyperpigmentation of knuckles, oral mucosa and rarely Addisonian pigmentation have been described. In older children, isolated anemia or bicytopenia with subtle macrocytosis may delay diagnosis. Severe presentations, seen in approximately 10% of cases, include pancytopenia, hemolytic anemia, and pseudothrombotic microangiopathy, which may mimic hematological malignancies or thrombotic microangiopathies. Early recognition is crucial, as vitamin B12 replacement leads to rapid and often complete hematological recovery.

## 1 Introduction

Vitamin B12 has an important role in the cellular metabolism, especially in the synthesis of deoxyribonucleic acid (DNA), methylation and mitochondrial metabolism. Since structurally, vitamin B12 is a planar macrocyclic ring composed of four pyrrole rings with cobalt at its center, it is also called as cobalamin (1). Methylcobalamin and 5-deoxyadenosylcobalamin are the metabolically active forms of vitamin B12, while two others forms–hydroxycobalamin and cyanocobalamin–become biologically active after they are converted to methylcobalamin or 5-deoxyadenosylcobalamin.

Cobalamin serves as a cofactor for two enzymes that have important biological functions (2). Methylcobolamin is a cofactor for methionine synthase, an enzyme involved in the conversion of homocysteine to methionine, which ultimately generates folate (vitamin B9). Folate is required to make thymidine, a fundamental building block of DNA. Without vitamin B12, folate becomes “trapped,” stunting DNA production in rapidly dividing cells, like those in the bone marrow (3). The enzyme methylmalonyl-CoA-mutase requires adenosylcobalamin as a cofactor, and converts L-methylmalonyl-CoA into succinyl-CoA which is needed for myelin synthesis (4). Consequently, vitamin B12 deficiency presents with features to ineffective erythropoiesis or abnormal myelination.

## 2 Requirements

Vitamin B12 is an essential water-soluble micronutrient that needs to be taken as a dietary supplement as humans cannot produce this vitamin. Foods of animal source are the only natural source of cobalamin in human diet. These include meats, fish, eggs, and dairy products. The recommended daily intake of cobalamin is 0.4 mg for the first 6 months of life and 0.5 mg for 6–12 months, 0.9 mg for 1–3 years, 1.2 mg for 4–8 years, 1.8 mg for 9–13 years, and 2.4 mg for 14 years through old age. In pregnancy, a daily intake of 2.6 mg is recommended, and in lactation, 2.8 mg (5). Since, human body can store vitamin B12 and these stores may last up to 2–5 years, most manifestations develop gradually.

## 3 Etiopathogenesis of vitamin B12 deficiency

**FIGURE 1:**
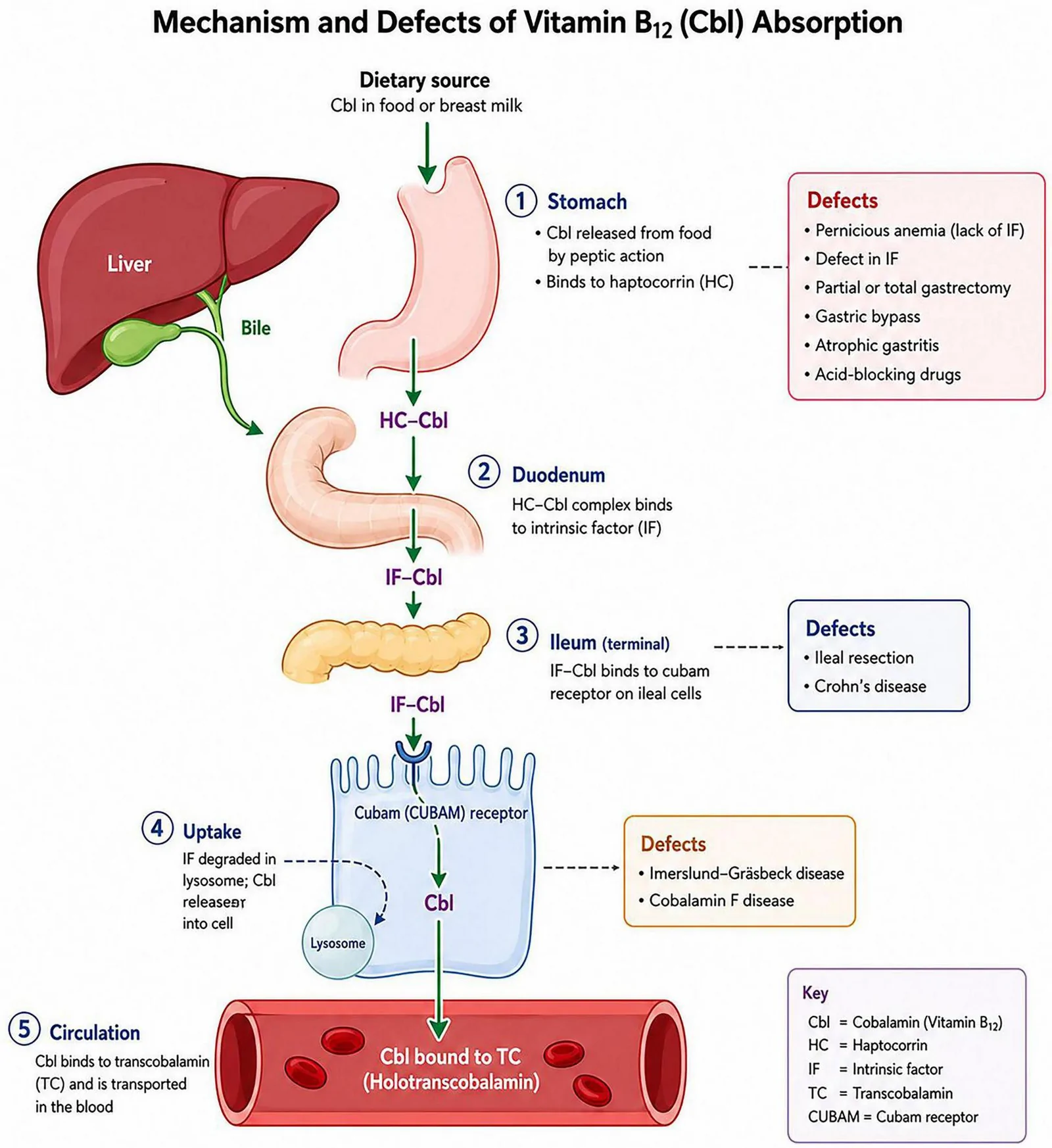
The normal absorption of vitamin B12 and the associated defects.

Vitamin B12 is predominantly derived from animal food sources and its deficiency is common in vegetarians and those with malabsorption. Dietary vitamin B12 is protein-bound and must be released before it is absorbed. The saliva as well as hydrochloric acid in stomach and gastric proteases help to free the vitamin B12 which then binds with haptocorrin (HC), also called as transcobalamin-1 or cobalophilin, a glycoprotein that binds and transports vitamin B12 (cobalamin). In the duodenum, this freed vitamin B12 combines with intrinsic factor (IF), a transport and delivery binding protein secreted by the stomach’s parietal cells and this complex is absorbed in the distal ileum by cubum receptor-mediated endocytosis. Further, the cellular lysozymes dissociate IF from vitamin B12 which circulates in the body bound to transcobalamin (Holotranscobalamin). Figure 1 illustrates the physiology of absorption of dietary or oral supplemental vitamin B12.

Thus, defects in intrinsic factor (IF) secretion, reduced gastric hydrochloric acid production, impaired absorption in the distal ileum, or abnormalities in vitamin B12 transport proteins may contribute to deficiency. The various etiologies of vitamin B12 deficiency are summarized in Table 1. Inadequate dietary intake remains the most common cause. Since plant-based diets lack reliable sources of vitamin B12, individuals following vegetarian diets and infants born to vegan/vegetarian mothers are particularly vulnerable. Furthermore, in many low- and middle-income countries, inadequate consumption of animal-source foods, including meat and poultry, may result in insufficient vitamin B12 intake even among non-vegetarian populations (6, 7).

**TABLE 1:** Etiological classification of vitamin B_12_ deficiency by physiological mechanism and clinical severity.

Category	Associated diseases/conditions	Clinical severity
Dietary deficiency (inadequate intake)	1. Adults: vegans, strict vegetarians, low meat/dairy diet, alcoholics, elderly 2. Infants: breastfed by vitamin B12-deficient mothers	Mild to severe
Food-bound malabsorption (failure to liberate from food)	1. Protein-bound malabsorption 2. Mild, non-immune, chronic atrophic gastritis 3. Medications: metformin, proton pump inhibitors, blockers 4. Chronic pancreatic disease (impaired haptocorrin degradation)	Mild to moderate
Competitive consumption of vitamin B12 (54)	1. Bacterial overgrowth 2. Infestation of small intestine by *Diphyllobothrium latum*	Mild to moderate
Intrinsic factor (IF)-cubam axis failure (complete loss of active absorption)	1. Pernicious anemia (autoimmune gastritis) 2. Gastrectomy/gastric bypass/bariatric surgery 3. Ileal resection/ileal conduit/ileocystoplasty 4. Inherited defects in IF or cubam receptor: Imerslund-Gräsbeck syndrome	Severe
Chemical inactivation	1. Nitrous oxide abuse (oxidizes and inactivates) (55)	Severe
Inherited metabolic defects	1. Transcobalamin (TC) deficiency 2. Inborn errors of cobalamin metabolism	Severe

## 4 Laboratory manifestations

Impaired DNA synthesis due to vitamin B12 deficiency, has a significant impact on hematopoiesis as it causes disruption of rapidly dividing hematopoietic precursor cells (8). Vitamin B12 deficiency leads to arrested nuclear division without significant alteration in the cytoplasmic maturation cycle leading to oversized but dysfunctional red blood cells, referred to as “nucleocytoplasmic (NC) dissociation” or “NC dyssynchrony” in the growing blood cells. All formed blood elements are affected by the ineffective megaloblastic hematopoiesis, but erythrocytes show the most marked changes, both in size and in shape, with large oval macrocytes and prominent anisopoikilocytosis. The spectrum of biochemical and hematological manifestations is as follows:

### 4.1 Hematological features

**FIGURE 2:**
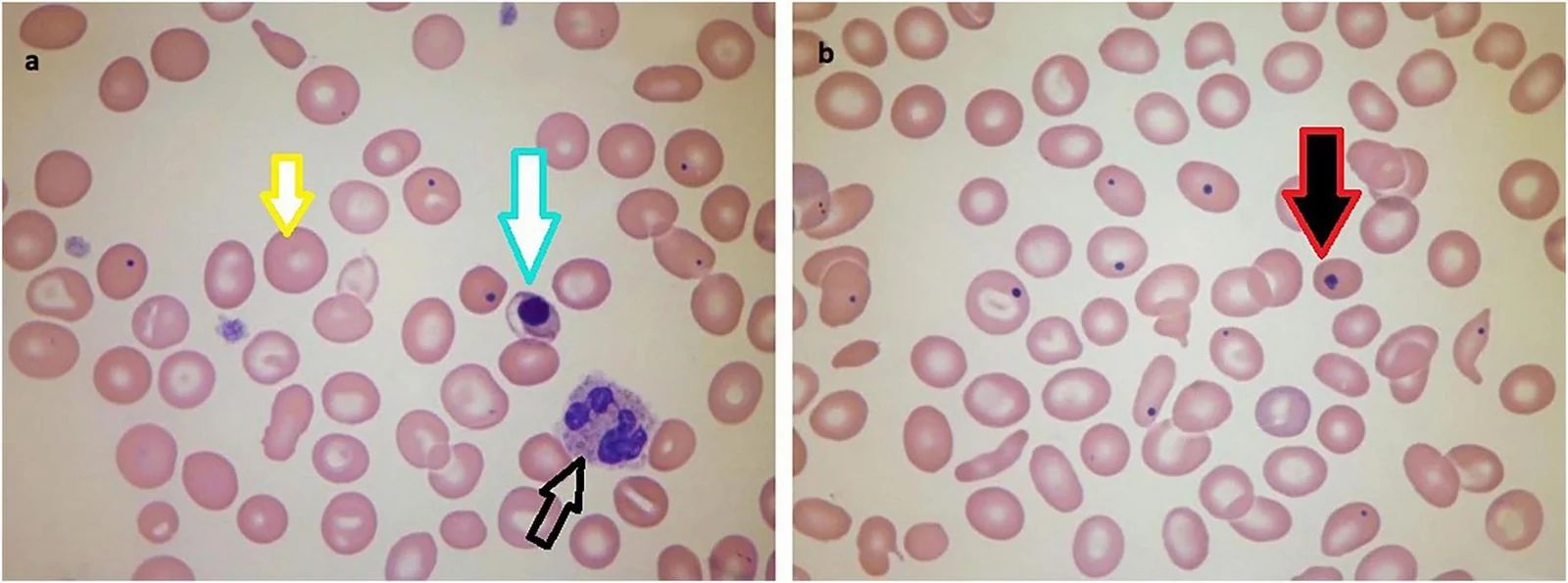
**(a)** Peripheral smear showing hypersegmented neutrophil (black arrow), macrocytes (yellow arrow), nucleated red blood cell (green arrow). **(b)** Peripheral smear showing Howel Jolly bodies (red arrow).

(1). Macrocytic anemia is the most common manifestation of vitamin B12 deficiency (Figure 2a). Macrocytosis, i.e., red blood cells (RBCs) larger than normal, indicated by a raised mean corpuscular volume (MCV) for age is a classic hallmark of vitamin B12 deficiency. Red blood cells in the neonatal period are physiologically macrocytic. Post neonatal period, the upper limit of MCV decreases to 85 fL. Between 6 months to 10 years of age, the upper limit of MCV is calculated by the following formula: MCV (fL) = 84 + [0.6 × Age (y)] (9). Between 10–18 years, the upper limit of MCV to diagnose macrocytic anemia is 90 fL while in adults the cut-off is 100 fL. Early in the disease, macrocytosis may be subtle as the average cell size shifts gradually due to the mixing of new, enlarged red cells with the existing, normocytic population. As the condition progresses, anisocytosis becomes more pronounced. An increase in red cell distribution width (RDW) is often the earliest measurable change in red cell indices. This is accompanied by a low reticulocyte count.

**FIGURE 3:**
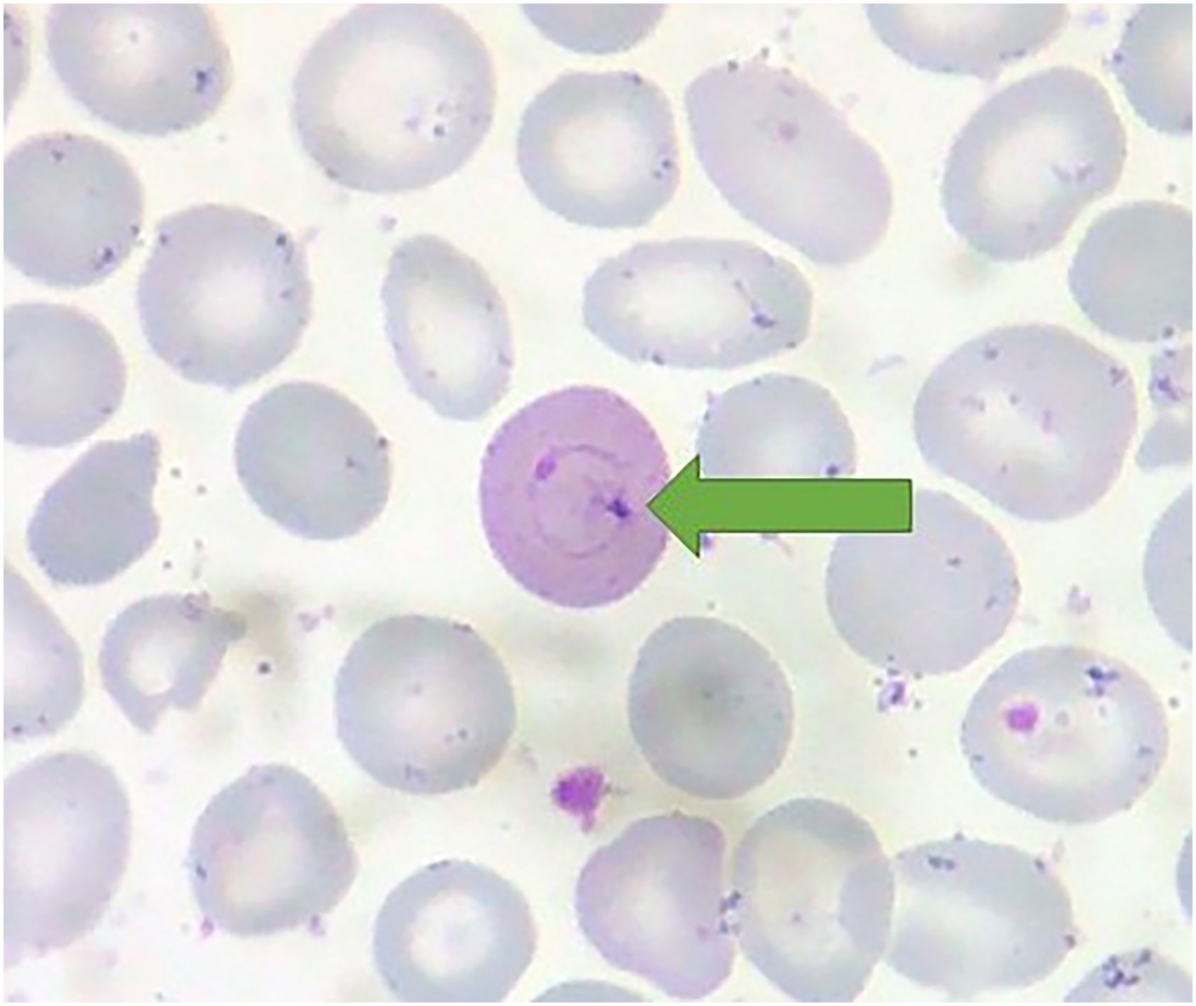
Peripheral smear showing Cabot rings (green arrow) in the red blood cells.

**FIGURE 4:**
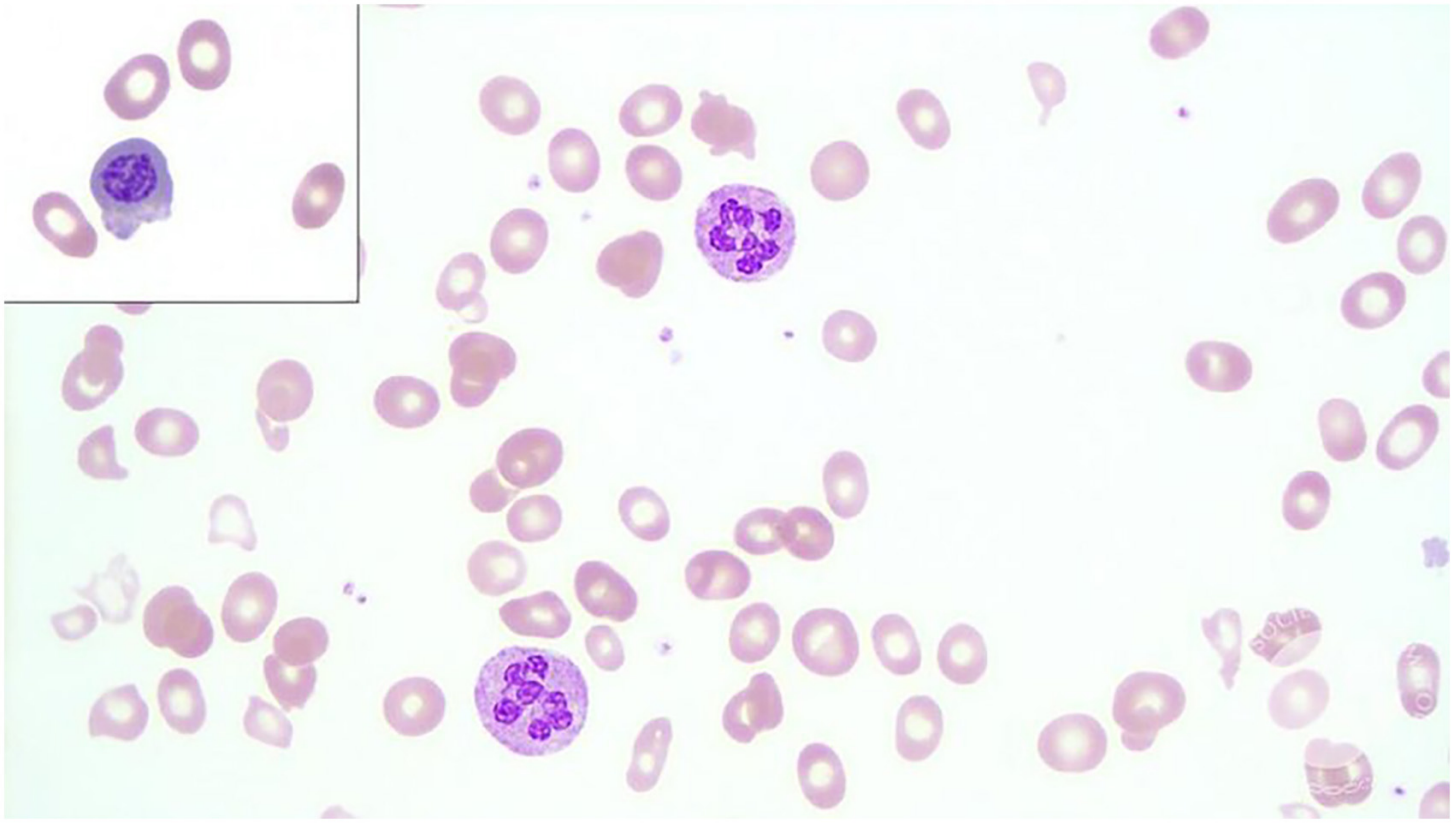
Peripheral smear showing a macropolycyte and circulating megaloblast (inset).

About 25% patients can have masked macrocytosis due to the presence of concurrent iron deficiency or alpha/beta-thalassemia traits which can result in a normocytic blood picture that can delay the diagnosis (10).

(2). White blood cell abnormalities: The presence of hypersegmented neutrophils (defined as >5% neutrophils with >5 lobes, or any neutrophil with six or more lobed nucleus) on the peripheral blood smear is the hallmark of vitamin B12 deficiency (11) (Figure 2a). Occasionally, macropolycytes (i.e., very large neutrophils with up to 8 or more nuclear segments) may also be seen. Segmented neutrophils are an early sign of megaloblastosis and usually persist for 10–14 days after treatment (12). Reduced production and accelerated intramedullary destruction of erythroid precursors also causes a drop in absolute neutrophil (neutropenia) and leukocyte counts (leucopenia).

(3). Thrombocytopenia is also not uncommonly observed as a consequence of increased intramedullary destruction and ineffective thrombopoiesis. Platelets so produced may be functionally abnormal.

(4). Peripheral smear typically shows macro/maco-ovalocytes with hypersegmented neutrophils along with a low reticulocyte count. It may show the presence of Howell Jolly bodies (small, dark-purple nuclear DNA fragments in RBCs) (Figure 2b), Cabot rings (mitotic spindle remnants appearing as loop or figure of 8, red-violet staining strands found in RBCs) (Figure 3), and punctate basophilia. Megaloblasts can be seen in the peripheral smear in patients with severe anemia (Figure 4). The abnormal red blood cells in peripheral smear and the megaloblastic precursors in peripheral blood are particularly vulnerable to hemolysis and therefore the peripheral blood picture may show features of hemolysis. Immaturity across multiple lineages in bone marrow can occasionally push nucleated red blood cells (nRBCs) and early myeloid precursors (metamyelocytes, myelocytes) into peripheral circulation, mimicking primary myelofibrosis or marrow infiltration (leukoerythroblastosis). The morphologic features of megaloblastic anemia are often significantly exacerbated in patients who are splenectomised or have functional asplenia, such as those with celiac disease or sickle cell anemia.

**FIGURE 5:**
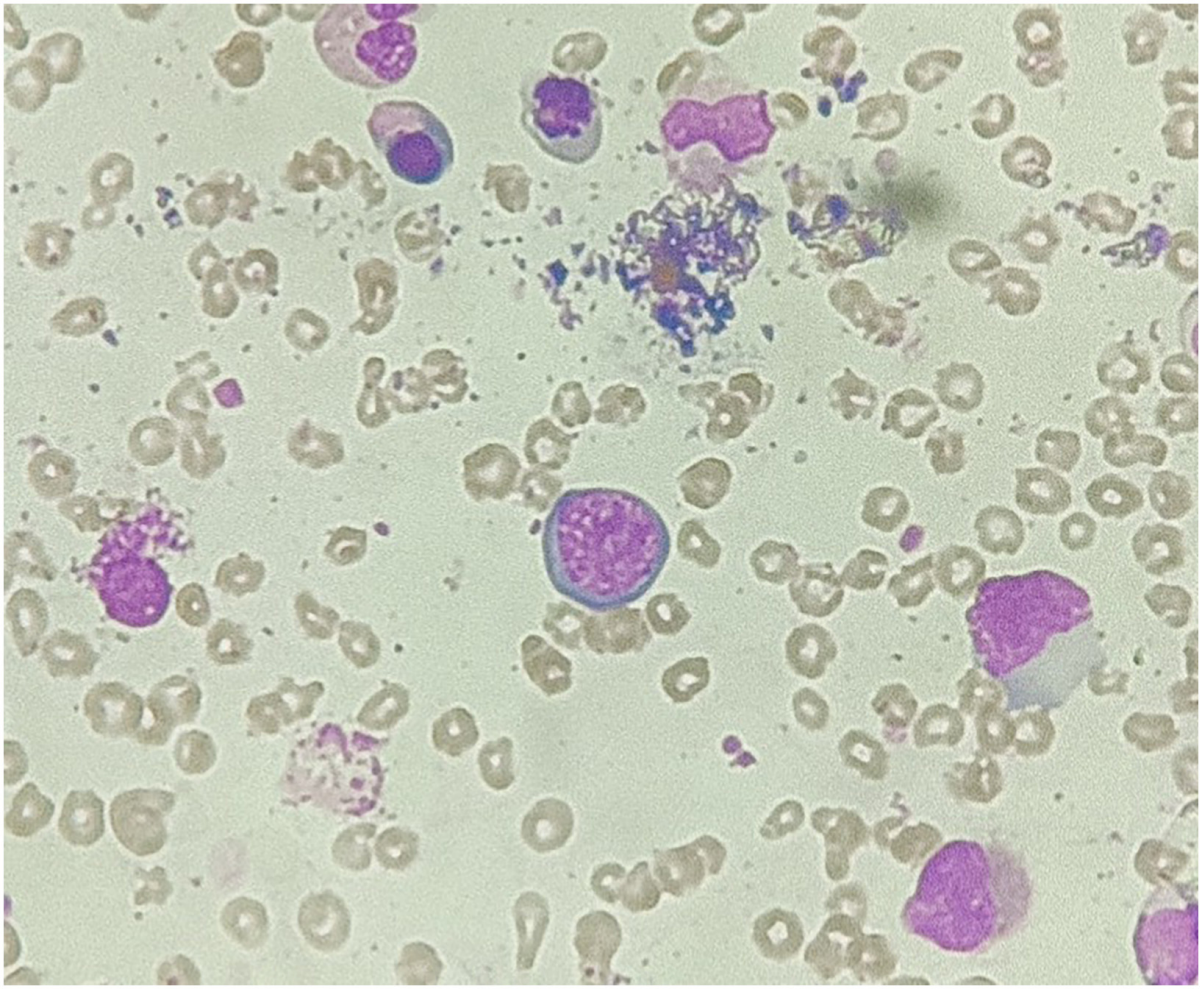
Bone marrow aspirate showing megaloblast in the centre.

**FIGURE 6:**
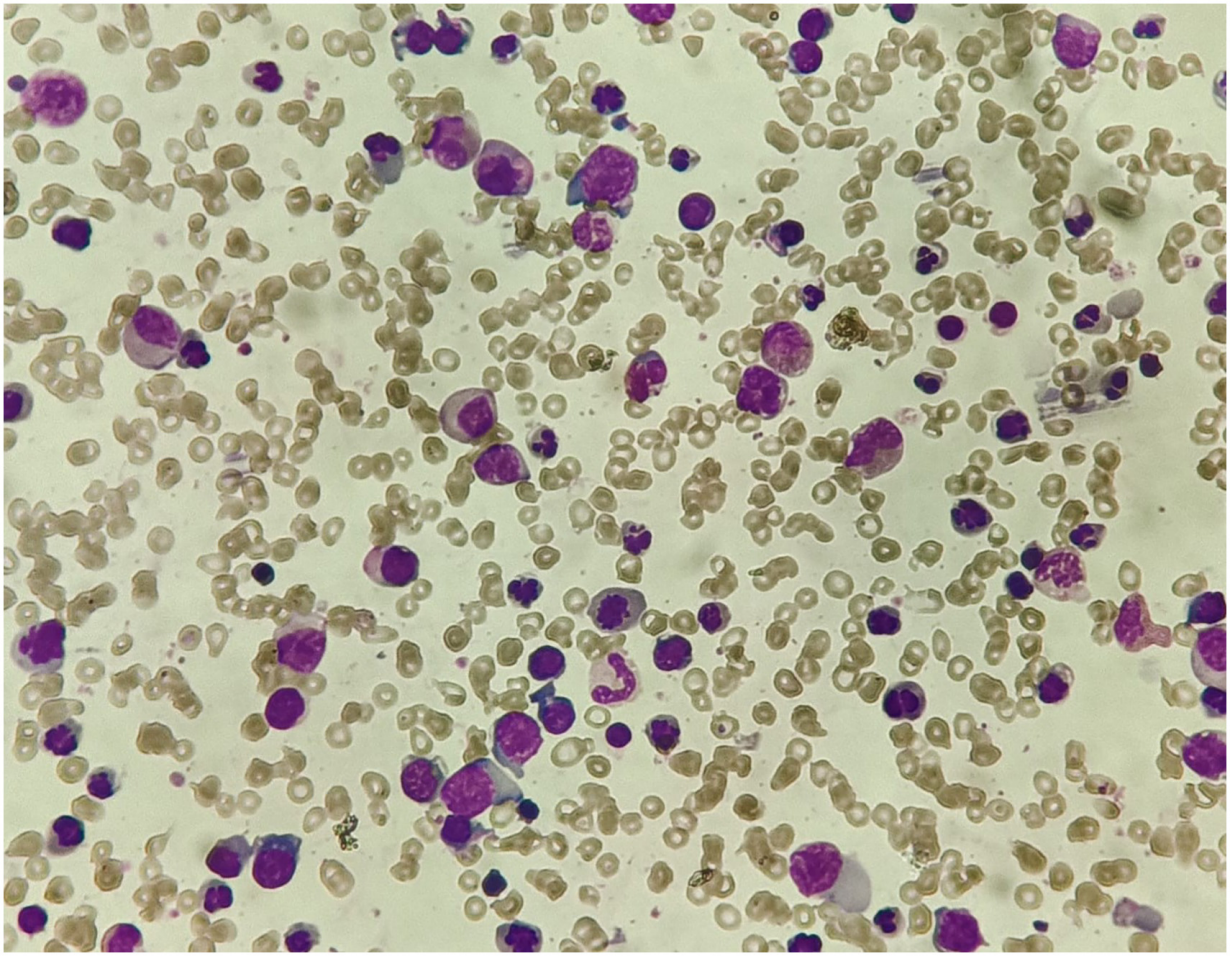
Bone marrow aspirate showing erythroid hyperplasia and marked dyserythropoiesis.

In profound, chronic states of vitamin B12 depletion, marrow production failures extend beyond red blood cells and pancytopenia may be observed (13). Although, it is a late finding, it is relatively common in pediatric cohorts with nutritional vitamin B12 deficiency. Thus, vitamin B12 deficiency is a close mimicker of aplastic anemia in absence of hepatosplenomegaly or acute leukemia when associated with hepatosplenomegaly. The megaloblastic anemia must be considered a crucial differential diagnosis for individuals with pancytopenia.

(5). Bone marrow abnormalities: Though bone marrow aspiration or biopsy is not typically needed for the diagnosis of B12 deficiency, but many patients with severe pancytopenia or atypical clinical features should undergo this procedure to exclude leukemia, myelodysplastic syndrome or aplastic anemia based on clinical suspicion. Nucleated erythrocytic precursor cells in the bone marrow develop immature or morphologically abnormal nuclei (megaloblasts), giant metamyelocytes and band cells. Megaloblasts are large erythroid precursors with an immature nucleus with open, sieve-like chromatin (Figure 5). Prolonged vitamin B12 deficiency leads to intramedullary hemolysis of the developing erythropoietic precursor cells in the bone marrow with compensatory hypercellularity (Figure 6). Bone marrow is markedly hypercellular with erythroid hyperplasia and reversal of M:E ratio (1:1 or 1:3 or lower). The erythroid cells show large nuclei with open reticular or lacey chromatin despite differentiation of the cytoplasm. It shows megaloblastic changes with some megaloblasts showing Howell-Jolly bodies and chromatin stippling. Other features of dyserythropoeisis including nuclear budding, irregular nuclear membrane, nuclear fragments, etc., may also be seen. Granulocytic precursors demonstrate giant metamyelocytes and band forms, while megakaryocytes appear enlarged with hyperlobulated or abnormal nuclei and reduced cytoplasmic granulation. Neutrophil hypersegmentation often precedes anemia. Megakaryocyte abnormalities are the least common change and megakaryocytes show nuclei with open chromatin and nuclear lobular hyper segmentation.

Prussian blue staining of the bone marrow aspirate may not always differentiate vitamin B12 deficiency from myelodysplastic syndrome (MDS), an important diagnostic mimic as sideroblasts may be seen in both. The presence of ring sideroblasts (where iron encircles the nucleus) in quantities > 15% is a defining characteristic of specific MDS subtypes. Also, unlike MDS, these changes usually resolve following vitamin B12 replacement. Bone marrow findings in vitamin B12 deficiency can also closely mimic hematological malignancies. Unlike megaloblasts (erythroid lineage) which have an open, lacy chromatin and mature cytoplasm, leukemic blasts (lymphoblasts in lymphoblastic leukemia and myeloblasts in myeloid leukemia) have fine chromatin with prominent nucleoli and comprise ≥ 20% of the cells in marrow.

Marked hypercellularity, prominent nuclear fragmentation (karyorrhexis), numerous mitotic figures, and an increased proportion of early promegaloblasts may be mistaken for myelodysplastic syndrome (MDS) or acute myeloid leukemia (AML), potentially leading to diagnostic confusion.

(6). Flow cytometry: The bone marrow aspirates may show a marked expansion of the CD34 population of hematopoietic precursors and CD235A (glycophorin-A) in erythroblasts which may be misinterpreted as indicating MDS or even acute leukemia, especially pure erythroid leukemia (14). There is a heterogeneity in the expression of CD36 and CD71 akin to MDS.

(7). Cytogenetics abnormalities: Vitamin B12 deficiency can also lead to cytogenetic changes which may be non-specific and show elongated and broken chromosomes, changes that are usually corrected within 2 days of treatment, although some abnormalities may remain for months (15).

### 4.2 Biochemical evaluation

(1). Vitamin B12 (cobalamin) assay is the preferred first-assay to confirm the vitamin B12 status, especially if the peripheral smear findings with a low reticulocyte count. Levels < 200 pg/mL (<148 pmol/L) are suggested to define vitamin B12 deficiency (WHO cut-offs) (16). Levels between 200 and 300 pg/mL (148–221 pmol/L) are considered borderline and warrant additional testing- e.g., Methyl malonic acid (MMA), Homocysteine (Hcy). Serum vitamin B12 levels > 300 pg/mL (>221 pmol/L) are traditionally considered within the normal range and generally exclude significant vitamin B12 deficiency. However, some individuals may remain functionally deficient at cellular level despite having serum B12 concentrations above this threshold. Therefore, in patients with clinical features suggestive of vitamin B12 deficiency but normal serum B12 levels, metabolic defects affecting intracellular uptake, transport, or utilization of vitamin B12 should be considered. It is also important to recognize that serum vitamin B12 testing has limited specificity and may be influenced by recent dietary intake.
(2). Holotranscobalamin (Holo TC) assay has better sensitivity and specificity in identifying B12 deficiency than serum vitamin B12 assays. The expected values for HoloTC in healthy individuals are 35–171 pmol/L. A cut-off of less than 35 pmol/L is suggested for defining deficiency (16). Though it has been used in studies, it is not available widely in India.
(3). Serum methylmalonic acid (MMA) assay is a sensitive functional marker of intracellular vitamin B12 deficiency and is useful when serum vitamin B12 levels are inconclusive. Vitamin B12 is a cofactor in the conversion of methylmalonyl-CoA to succinyl-CoA and a deficiency of vitamin B12 leads to elevated levels of MMA. Elevated MMA levels reflect impaired B12 metabolism, making it a critical tool for early detection and intervention. The normal range of plasma MMA is 70–270 nmol/L and the references are laboratory dependent and hence the results should be interpreted carefully. Exceptionally high levels of plasma MMA (750 nmol/L) almost invariably indicate vitamin B12 deficiency (16).
(4). Serum homocysteine (Hcys) levels are also considered a functional marker of vitamin B12 deficiency and should be obtained in patients with clinical suspicion of vitamin B12 deficiency but borderline serum levels. Both vitamin B12 and folate are required for the metabolism of homocysteine to methionine, and hence deficiency of either can lead to accumulation and elevated levels of homocysteine. If MMA is normal and homocysteine is raised, folate deficiency would be more likely. Increased methylmalonic acid (MMA) levels are widely considered to be a highly sensitive and specific test for identifying vitamin B12 deficiency. The British Society of Hematology suggests Hcys above 15 mol/L could be indicative of folate or vitamin B12 deficiency (16).
(5). Serum folate assay: Both folate and vitamin B12 deficiency can cause macrocytic anemia, megaloblastic bone marrow changes and hypersegmented neutrophils. Measuring serum folate helps distinguish isolated vitamin B12 deficiency from folate deficiency or combined deficiencies. This also has therapeutic implications as folate therapy may correct the anemia of vitamin B12 deficiency while allowing neurological damage to progress. Therefore, identifying vitamin B12 deficiency before administering folate is important. Serum folate is often normal or elevated in vitamin B12 deficiency because of the “methyl-folate trap,” in which folate becomes trapped as 5-methyltetrahydrofolate due to impaired methionine synthase activity. Thus, in patients with macrocytic anemia, vitamin B12 and folate are often measured together. If vitamin B12 levels are borderline, measurement of homocysteine (elevated in both deficiencies) and MMA (elevated only in vitamin B12 deficiency) can help differentiate the two conditions. Serum folate levels < 3 ng/mL (6.8 nmol/L) are used to signify folate deficiency in a patient with macrocytic anemia. Using homocysteine concentrations as a metabolic indicator, the cut-off value for determining folate deficiency in all age groups is defined as serum or plasma folate lower than 4 ng/mL (10 nmol/L) (17).
(6). All the biochemical markers of blood cell destruction may be present such as elevated indirect bilirubin, decreased haptoglobin, increased transaminases, and elevated lactate dehydrogenase (LDH) because of the intramedullary destruction of erythrocytic precursors (18, 19).
(7). Transferrin saturation and ferritin will increase unless there is coexisting iron deficiency anemia (20, 21).
(8). Occasional patients will have signs of malnutrition such as low serum albumin, hypogammaglobulinemia, and malabsorption of other vitamins.
(9). Tests for pernicious anemia: Anti-intrinsic factor antibodies have a high positive predictive value (95%) and have a high specificity (98%–99%) (16). However, sensitivity is only 40-60% as seen in studies amongst adults. Data in children is lacking. *Gastric parietal cell antibodies* have a sensitivity of 80% for diagnosis of pernicious anemia. However, it is positive in 10% normal individuals as well (16). Hence, positive antibody is not definitive for pernicious anemia. Routine testing of anti-gastric parietal cell or intrinsic factor antibody is not recommended.
(10). Tests for genetic disorders or inborn errors of metabolism causing megaloblastic anemia: Disorders affecting uptake and metabolism of vitamin B12, as well as thiamine responsive megaloblastic anemia, orotic aciduria, and 3-phosphoglyceride dehydrogenase deficiency, may also cause megaloblastic anemia. These conditions warrant evaluation for specific mutations and specific biochemical assays which are beyond the scope of this review.

## 5 Clinical manifestations

The clinical manifestations of vitamin B12 deficiency can be varied and range from asymptomatic to severe hematological manifestation (*vide supra*) or varied neurological presentations including developmental delay in infants, hypotonia, neuropathy, tremors, seizures, and myelopathy. Some of the lesser-known manifestations are cutaneous such as skin hyperpigmentation, stomatitis, hair and nail changes, and pseudo-thrombotic microangiopathy and thrombotic events (22). Hence, vitamin B12 deficiency is now recognized as a “great masquerader” presenting with a variety of non-specific symptoms (23). Herein, we will focus on the diagnostic paradigms characterizing the hematological presentation of cobalamin deficiency.

(1). Anemia is the most common clinical presentation of vitamin B12 deficiency. Usually, anemia develops gradually, and is mild; majority present with a sallow complexion and non-specific features like weakness, lassitude, low grade fever, and poor growth. Shortness of breath (primarily with exertion), palpitations, cardiac decompensation and light headedness are encountered in patients with severe anemia. Physical examination may reveal pallor, mild icterus, tachycardia, functional cardiac murmurs, and beefy tongue (Moeller-Hunter glossitis or atrophic glossitis characterized by a smooth, glossy, and often bright red, painful tongue with complete atrophy of lingual papillae).
(2). Mild jaundice: Patients can develop mild jaundice due to both intramedullary and extravascular hemolysis. The combination of severe pallor with jaundice caused by hemolysis may result in a characteristic lemon-yellow hue of the skin.
(3). Hepatosplenomegaly: Vitamin B12 deficiency can lead to mild to moderate hepatosplenomegaly due to extramedullary hematopoiesis following hemolysis.
(4). Susceptibility to infections: Leukopenia predisposes these patients to repeated infections.
(5). Pseudo-thrombotic microangiopathy (pseudo-TMA): Vitamin B12 deficiency–associated pseudo-TMA is a rare but important hematological manifestation of severe cobalamin deficiency that clinically mimics thrombotic microangiopathy (24). It is characterized by hemolytic anemia, thrombocytopenia, and the presence of schistocytes on peripheral smear, often resembling conditions such as thrombotic thrombocytopenic purpura (TTP). However, unlike true thrombotic microangiopathy, the underlying mechanism is ineffective erythropoiesis and intramedullary hemolysis resulting from impaired DNA synthesis. Markedly elevated lactate dehydrogenase levels, low reticulocyte count, macrocytosis, and severe vitamin B12 deficiency help distinguish pseudo-TMA from TTP. Vitamin B12 deficiency also causes an increase in homocysteine levels, which leads to endothelial dysfunction and results in fragmentation of erythrocytes to schistocytes. Endothelial dysfunction contributes to vasoconstriction, increased platelet aggregation, and abnormal activation of the coagulation causing the clinical features of microangiopathy. Early recognition is crucial, as treatment with vitamin B12 replacement leads to rapid hematological recovery and avoids unnecessary interventions such as plasma exchange.
(6). Bleeding diathesis: Patients may present with petechiae, ecchymoses, or mucosal bleeding due to isolated thrombocytopenia or pancytopenia. In rare cases, severe hemorrhagic manifestations have been reported in megaloblastic anemia, resulting from profound thrombocytopenia and associated platelet dysfunction. Such presentations may mimic primary hematological malignancies or immune thrombocytopenia (ITP), leading to diagnostic confusion.
(7). Thrombosis: The deficiency of vitamin B12 can be one of the uncommon causative factors of thrombophilia. This predisposition is related to the development of hyper homocysteinemia, although other concurrent factors can participate in specific cases. An accumulation of excess homocysteine in the absence of sufficient vitamin B12 leads to platelet activation via thromboxane A2 biosynthesis, endothelial damage and dysfunction, as well as activation of protein C via activation of factor Va leading to an increased incidence of thrombosis including deep vein thrombosis and cerebral venous thrombosis (25–27).
(8). Clinical mimicry: Severe pancytopenia accompanied by hypersegmented neutrophils in peripheral smear can closely resemble myelodysplastic syndromes (MDS) or aplastic anemia or acute leukemia, requiring careful diagnostic differentiation including bone marrow aspiration (28).

### 5.1 Age-specific hematological manifestations

Traditionally considered a disease of older adults with pernicious anemia, vitamin B12 deficiency is now recognized as a lifespan disorder, affecting infants, children, and adolescents, pregnant women and the elderly, with varying manifestations based on physiological demand and metabolic context. Understanding the age-specific variations in these presentations is critical for early diagnosis and the prevention of irreversible neurological and hematological sequelae. See Table 2.

**TABLE 2:** Age-wise etiologies and clinical manifestations of vitamin B12 deficiency.

Age-group	Primary etiology	Dominant clinical features
Infants	Maternal deficiency, weaning patterns	Failure to thrive, developmental regression, hypotonia, infantile tremors.
Toddlers/kids	Nutritional (strict vegetarianism)	Anemia (often severe), glossitis, stomatitis, impaired scholastic performance.
Adolescents	Dietary fad, inflammatory bowel disease	Fatigue, glossitis, occult neurological symptoms.
Adults	Autoimmune (Pernicious anemia), *H. pylori* infection	The “Classic Triad”: anemia, jaundice, glossitis
Elderly	Atrophic gastritis, drug-induced (proton pump inhibitors, metformin)	Cognitive decline, subacute combined degeneration, neuropathy.

#### 5.1.1 Infants and young children (0–5 years)

Infants are particularly vulnerable to vitamin B12 deficiency and may present with severe hematological and neurological manifestations including feeding difficulties, regurgitation, constipation, and varying neurological symptoms like apathy, irritability, paresthesias, hypotonia, seizures, sensory deficits, developmental delay or regression, infantile tremor syndrome, or cognitive delay (29). Low maternal vitamin B12 status, prolonged breastfeeding and a low intake of animal food after weaning are major risk factors for vitamin B12 deficiency in infancy. Studies from India have reported a vitamin B12 deficiency prevalence of up to 65% among such infants (30, 31), whereas studies from Western countries have reported rates of up to 40% (32).

Uncommon causes of vitamin B12 deficiency in infants that need evaluation include inborn errors of cobalamin metabolism and Imerslund-Gräsbeck Syndrome (IGS). IGS, a rare autosomal recessive disorder characterized by selective intestinal malabsorption of vitamin B12 despite normal secretion of intrinsic factor and gastric acid, typically presents in childhood with failure to thrive, pallor, fatigue, recurrent infections, and occasionally mild neurological manifestations. Laboratory findings include megaloblastic anemia, often accompanied by low-grade proteinuria. IGS results from biallelic pathogenic variants in the *CUBN* or *AMN* genes, which encode cubilin and amnionless, respectively. These proteins form the cubam receptor complex in the terminal ileum, which is essential for intrinsic factor–vitamin B12 uptake and renal tubular protein reabsorption. Consequently, affected individuals develop vitamin B12 deficiency and may exhibit persistent proteinuria. Other genetic causes of vitamin B12 deficiency like transcobalamin II deficiency intrinsic factor deficiency, and intracellular cobalamin metabolic defects (particularly cblC disease) must be considered in infants presenting with varied manifestations including failure to thrive, hypotonia, metabolic acidosis and neurocognitive defects (33, 34).

#### 5.1.2 School-age children (5–12 years)

A study conducted in North India on 111 children, with median age 9 years, showed a vitamin B12 deficiency prevalence of 64.8% (37). In school-aged children, the manifestations may be more gradual, often presenting as mild to moderate macrocytic anemia with a classical triad of red cell indices [elevated MCV, low mean corpuscular hemoglobin (MCH), and low mean corpuscular hemoglobin concentration (MCHC)], though co-existing iron deficiency can sometimes mask macrocytosis. Dermatological manifestations like hyperpigmentation of knuckles, icterus and angular stomatitis, brittle hair, nail changes, icterus and commonly seen in children with underlying vitamin B12 deficiency. These may occur even before the development of hematological and neurological complications and hence these findings may aid early diagnosis. Lower school performance, reduced weight, height and head circumference, impaired mental and social development including affliction of short-term memory and attention warrant careful evaluation for underlying vitamin B12 deficiency.

#### 5.1.3 Adolescents (up to 18 years)

The etiology of vitamin B12 deficiency in adolescents represents a unique epidemiological shift. The underlying causes include dietary insufficiency, inflammatory and structural enteropathies, juvenile autoimmune pathologies like juvenile pernicious anemia, late-manifesting or subclinical inherited disorders. Common symptoms include lethargy, easy fatigability, and anorexia. Physical signs may include pallor with a sallow complexion and glossitis (inflammation of the tongue). A study involving 200 adolescents indicated that folate and vitamin B12 deficiencies were more prevalent than iron deficiency (36). Another study of 40 adolescents with severe anemia found megaloblastic anemia to be the most common type (42.5%) (37).

#### 5.1.4 Adult and geriatric population

The etiologies driving cobalamin deficiency in adults can be systematically categorized into four primary pathophysiological mechanisms: autoimmune destruction, malabsorption, dietary insufficiency, and drug-induced interference. Etiology shifts towards malabsorptive processes. Pernicious anemia, chronic atrophic gastritis, and drug intake (proton pump inhibitors, metformin) remains a dominant cause in this age-group. Vitamin B12 deficiency is particularly difficult to identify in elderly individuals because the typical hematological and neurological manifestations are uncommon. They generally present with megaloblastic or macrocytic anemia, subacute combined degeneration of the spinal cord, impaired sensory and peripheral nerve function, Cognitive impairment, depression, bone disease, hearing loss and macular degeneration. Hematological symptoms occur in <50% of individuals, and, even when present, difficult to differentiate from other hematological disorders, such as myelodysplasia or aplastic anemia. Neuropsychiatric and neurological symptoms are more-frequently observed in elderly individuals who are vitamin B12 deficient than any other age group (38–40).

### 5.2 Other manifestations of underlying vitamin B12 deficiency

(1). Dermatological manifestations: The cutaneous manifestation of vitamin B12 deficiency includes hyperpigmentation, vitiligo, hair changes, and recurrent angular stomatitis. However, the most common dermatological manifestation is hyperpigmentation especially of knuckles and dorsal aspect of hands and feet. The darkening of sole and palmar creases, interphalangeal joints, and terminal phalanges has also been noted. Deficiency of vitamin B12 decreases the level of reduced glutathione, which activates tyrosinase and thus leads to transfer to melanosomes and defect in the melanin transfer between melanocytes and keratinocytes, resulting in pigmentary incontinence which remains the major factor of underlying hypothesis for hyperpigmentation in vitamin B12 deficiency (41).
(2). Mucocutaneous and gastrointestinal manifestations: Glossitis of the tongue, which is due to atrophy of tongue papillae, stomatitis, cheilitis and oral ulcers may occur in severe cobalamin or folate deficiency (42). Tissues with rapid turnover may also show megaloblastic changes such as intestinal villi resulting in malabsorption. Loss of appetite, nausea, vomiting, diarrhea, and unexplained weight loss may also be seen.
(3). Neurological manifestations: Vitamin B12 deficiency has a spectrum of neurological manifestations which are severe and warrant urgent parenteral therapy. These are especially seen in the inborn errors of cobalamin metabolism and are more common in young infants and toddlers. These are among the most debilitating and can become irreversible if left untreated. The spectrum includes developmental delay and learning difficulties, hypotonia, ataxia, tremors and seizures in children (43, 44). Older children and adults may have peripheral neuropathy, sensory (loss of vibratory and proprioception) and motor (weakness, hyperreflexia, spasticity) deficits, and features of subacute combined degeneration of the spinal cord due to demyelination in the posterior and lateral columns of the spinal cord. These are covered in detail in another article in this issue.
(4). Genital health and infertility: Vitamin B12 deficiency contributes to infertility by causing ovulatory dysfunction, poor egg quality, and defective endometrial implantation. It also affects cervical health, as low levels are associated with an increased risk of high-grade cervical lesions, and can lead to recurrent fetal loss (45). There may be decreased spermatogenesis or other fertility defects.

## 6 Treatment of hematological manifestations

Once the clinical features and laboratory findings corroborate to suggest cobalamin deficiency, treatment should be initiated with replacement therapy using vitamin B12 preparations without delay as timely treatment can reverse bone marrow failure and demyelination of nervous system. However, although treatment of vitamin B12 deficiency requires urgency, it is never an emergency and it should never preclude a diagnostic workup. However, if the child shows features of cardiorespiratory compromise due to severe anemia, blood transfusion may be given on an emergent basis before starting vitamin B12 replacement therapy. In case of discordance between laboratory results (normal serum cobalamin levels) and clinical features, treatment with vitamin B12 need not be delayed. Venous blood samples for estimation of complete hemogram and serum vitamin B12 (cobalamin), folate, holo-TC, MMA and HCys should be taken before starting therapy (46). Bone marrow aspiration to prove megaloblastic anemia is not necessary in all patients prior to starting treatment (8). It may be needed if there are atypical clinical features, or where there is a reasonable possibility of an alternate diagnosis like aplastic anemia or leukemia or MDS.

Parenteral (intramuscular or intravenous) vitamin B12 rapidly and reliably restores vitamin B12 stores and therefore intramuscular (IM) injections of vitamin B12 have been used as a standard treatment in vitamin B12 deficiency. Lately, there has been some evidence of using high-dose oral formulations in place of parenteral therapy given the ease of administration, low cost and convenience of using tablets and syrups in children. A Cochrane review including 153 adults concluded that oral and IM vitamin B12 routes have similar efficacy in terms of normalizing serum vitamin B12 levels in vitamin B12 deficiency (47).

Children with mild to moderate anemia, or non-neurological manifestations, can be treated with high dose oral cobalamin (22). Those with severe anemia may be preferably treated with IM cobalamin; treatment can be initiated with lower doses of oral vitamin B12 (25–50 μg) for the initial 2–3 days followed by 100 μg daily for the next 7 days, and thereafter by alternate day therapy over the next week and weekly doses over next 1 month. Monthly doses of vitamin B12 (1,000 μg/month) are administered thereafter over the next 2–3 months (22). The therapeutic response may be monitored by evaluating the reticulocyte count on day 7 and clinical improvement. Monthly complete blood counts and biochemical assessment of serum MMA and homocysteine may aid assessment of response.

The duration of therapy in these studies varied from 1 month to several years. A 1-month duration of oral therapy was found to be inadequate for hematological recovery (48, 49) as well as biochemical response in terms of normalization of serum cobalamin levels (49, 50). The vitamin B12 levels were found to plateau after 3 months of high dose oral therapy. Orally administered cobalamin should be taken in fasting state as food interferes with absorption of vitamin B12.

Recently, a few studies have shown alternate routes of vitamin B12 administration like sublingual route (51, 52) and intranasal (IN) route (53) which could offer the advantages of parenteral route i.e., bypassing the intrinsic factor for vitamin B12 absorption, may be useful in treating vitamin B12 deficiency.

Patients who fail to respond to therapy, must be evaluated for co-existing iron or folate deficiency or deficiency of other micronutrients. Those with malabsorption disorders need parenteral cobalamin therapy and also need to be evaluated for folate deficiency and treated with folic acid supplements (1–5 mg oral) as necessary.

## 7 Response to therapy

Clinical reversal: Clinical improvement with a sense of wellbeing starts within 24 h of treatment. Glossitis starts improving by day 2 and resolves by the end of the second week of treatment. The clinical evaluation can be scheduled within the first week for those with severe anemia and in the second week for children with moderate or mild anemia. Neurological improvement may take longer.

Laboratory recovery: Complete blood count (CBC) and MCV can be easily used to assess the response to therapy. The most useful objective parameter is the rise in reticulocyte count which starts within 48–72 h and peaks at the end of the first week after starting treatment; the reticulocytosis is usually proportional to the severity of the anemia. Normalization of MMA and plasma homocysteine levels occur in first 5 days, unless there is underlying renal dysfunction. White blood cell and platelet counts (if they had been low) normalize within 1 week. MCV begins to fall by day 14 and usually normalizes by week 6–8. Hypersegmented neutrophils disappear by 10–14 days of treatment. The complete blood counts, including mean corpuscular volume (MCV), should be completely normal by 6–8 weeks. Sustained normalization of serum cobalamin occurs following 2 weeks of therapy (8, 46)

## 8 Prevention

Prevention of vitamin B12 deficiency depends on ensuring adequate intake, identifying at-risk individuals, and providing appropriate supplementation when necessary. High-risk groups include those on vegetarian or vegan diets, elderly, those with pernicious anemia, short-bowel sundrome or underlying malabsorption (e.g., celiac disease, inflammatory bowel disease) and those who are on long-term medications associated with B12 deficiency (e.g., metformin, proton pump inhibitors). Individuals following strict vegetarian or vegan diets should consume B12-fortified foods or take vitamin B12 supplements. Maternal vitamin B12 intake during pregnancy and breastfeeding is important to prevent deficiency in exclusively breastfed infants. Vitamin B12 supplementation is needed for infants born to vitamin B12-deficient mothers. Periodic assessment of vitamin B12 status in high-risk individuals allows early identification and treatment before the development of anemia or neurological complications.
